# Mild dehydration does not alter acute changes in sweat electrolyte concentrations during exercise

**DOI:** 10.14814/phy2.16174

**Published:** 2024-09-18

**Authors:** Lindsay B. Baker, Michal Ozga, James R. Merritt, Shelby Alfred, Peter John D. De Chavez, J. Matthew Hinkley

**Affiliations:** ^1^ PepsiCo R&D Life Sciences Gatorade Sports Science Institute Valhalla New York USA; ^2^ Data Science & Analytics, PepsiCo R&D Plano Texas USA

**Keywords:** chloride, hydration, potassium, sodium, sweat biomarkers

## Abstract

The purpose of this study was to determine the effect of hydration status on the change in sweat sodium (Na^+^), chloride (Cl^−^), and potassium (K^+^) concentrations during exercise‐heat stress. Fifteen subjects (Six female, nine male; 29 ± 9 y; 71 ± 14 kg) completed 90 min of cycling (81% HR_max_) in the heat (~33°C, 42% rh) with fluid replacement to maintain euhydration (EUH) or without fluid to dehydrate to 2.4 ± 0.4% body mass loss (DEH). Sweat was collected from the forehead (FH), right scapula (SCAP), and left (LVFA) and right (RVFA) ventral forearms using the absorbent pad technique at the beginning (0–30 min) and end of exercise (60–90 min). Sweat was analyzed for Na^+^, Cl^−^, and K^+^ concentrations using ion chromatography. Data are reported as mean ± SD or median ± IQR. There were no differences (Paired *t*‐tests or Wilcoxon signed‐rank tests) between EUH and DEH in the change in sweat Na^+^ (FH: 24.3 ± 21.5 vs. 30.8 ± 22.4 mmol/L; SCAP: 9.7 ± 6.2 vs. 9.6 ± 8.2 mmol/L; LVFA: 7.5 ± 6.0 vs. 5.6 ± 5.9 mmol/L; RVFA: 8.2 ± 8.6 vs. 7.8 ± 5.2 mmol/L), sweat Cl^−^, or sweat K^+^ at any site (*p* = 0.07–0.99). The change in sweat electrolyte concentrations during 90 min of exercise in the heat was not significantly influenced by mild dehydration in recreational to moderately‐trained male and female athletes.

## 1 INTRODUCTION

In recent years, there has been significant interest in the potential of sweat as a biofluid for noninvasive diagnostics to track outcomes related to human health and performance (Clark & Ray, 2023; Yang, Ghaffari, & Rogers, 2023). A surge in sweat diagnostics research has been driven largely by advances in technology that has enabled on‐body collection and analysis of sweat composition. For example, previous research has shown that sensors of various form factors worn across the body (e.g., forehead, torso, arms) can collect and measure microliter volumes of sweat for real‐time analyses of electrolytes, metabolites, and other analytes (Brothers et al., 2019; Ehtesabi & Kalji, 2024; Gao et al., 2016; Salim & Lim, 2019).

One purported application for on‐body sweat analytics is real‐time monitoring of hydration status during exercise. Specifically, it has been suggested that changes in the concentrations of sweat electrolytes, such as sodium (Na^+^), chloride (Cl^−^), potassium (K^+^), or their ratios (e.g., Na^+^/K^+^) can serve as an indicator of dehydration (DEH) (Gao et al., 2016; Wang et al., 2022; Yang, Sun, et al., 2023). This is based, in part, on the finding that sweat Na^+^ concentration tends to increase (Gao et al., 2016; Yang, Sun, et al., 2023), and K^+^ concentration may decrease (Yang, Sun, et al., 2023) over time during exercise without fluid intake. If changes in sweat electrolyte concentrations are a good indicator of hydration status, then such wearables may potentially be useful in informing personalized fluid intake needs during exercise. However, these studies did not measure (or in some cases measured but did not show statistical results for) subjects' corresponding changes in sweat electrolyte concentrations while euhydrated (EUH) during exercise to compare with DEH trials in a crossover study design.

A few studies have compared the effects of DEH versus EUH on sweat composition during exercise (Armstrong et al., 1985; Morgan et al., 2004; Walsh et al., 1994). For example, Morgan et al. (2004) collected sweat from the dominant forearm of eight men during 120 min of cycling and found that sweat Na^+^ and Cl^−^ concentrations were higher in the trial where no fluid was consumed versus when subjects drank fluid to replace sweat losses. Armstrong et al. (1985) found that, after 6 h of exercise where 12 men were allowed to drink water of different temperatures ad libitum, DEH (1.0% and 2.1% body mass loss) was associated with lower whole body sweat Na^+^ and Cl^−^ concentrations than when there was minimal change in body mass (−0.5%). In another study, Walsh et al. (1994) used the arm bag technique to collect sweat from 6 men during 60 min of cycling exercise and found no difference in sweat electrolyte losses between DEH (1.8% body mass loss) and EUH. However, a limitation of these studies was that sweat was pooled into one sample for the full duration of exercise, and thus investigators were not able to compare changes from the beginning to end of exercise.

It has been suggested that since extracellular fluid is the precursor to primary sweat in the secretory coil of eccrine sweat glands, changes in sweat electrolyte concentrations could reflect DEH‐induced hemoconcentration (i.e., increases in blood Na^+^ and Cl^−^ concentrations) during exercise. However, differences in serum Na^+^ and Cl^−^ concentrations in DEH versus EUH trials (~3 mmol/L) reported previously are likely too small to cause a significant effect on sweat electrolyte concentrations (Morgan et al., 2004). Furthermore, time course changes in sweat electrolyte concentrations can also be influenced by other factors, such as local sweating rate (LSR). During exercise, local sweating initiates after reaching a body core temperature set point, and then LSR gradually increases until a steady state is reached approximately 20–30 min into activity (Boisvert et al., 1997; King et al., 2023). In turn, this ramp up in LSR is also associated with a gradual increase in sweat Na^+^ and Cl^−^ since there is a direct linear relation between LSR and the concentrations of sweat Na^+^ and Cl^−^ excreted in final sweat (Buono et al., 2008). On the other hand, a potential confounding factor regarding time course changes in sweat K^+^ concentration is skin surface contamination. For example, sweat K^+^ concentrations may appear higher in initial sweat samples due to their presence in the stratum corneum and subsequently decrease over time as surface contaminants are flushed away by further sweating (Ely et al., 2011; Verissimo et al., 2007).

To the authors' knowledge, no studies have compared the effects of DEH versus EUH on the change in sweat electrolyte concentrations along with corresponding LSR during exercise. Research on acute changes in sweat electrolyte concentrations during exercise is needed to help discern the effect of sample timing and LSR from the effect of hydration status per se. Therefore, the purpose of this study was to determine the effect of hydration status on the change in electrolyte concentrations during exercise‐heat stress. This study measured sweat Na^+^, Cl^−^, and K^+^ concentrations, sweat Na^+^/K^+^ ratio, and LSR at the beginning and end of a 90‐min exercise bout where male and female subjects maintained EUH or were dehydrated to 2–3% of body mass loss in a randomized counterbalanced crossover design. Sweat was collected at four different body sites to represent a broad range in LSR and sweat composition, and to assess if there are regional differences in the effect of hydration status on sweat electrolyte concentrations.

## 2 METHODS

### 2.1 Participants

Fifteen male (*n* = 9) and female (*n* = 6) recreational to moderately‐trained athletes (29 ± 9 y; 71 ± 14 kg) participated in this study. This study (clinical trial identifier: NCT06044610) was approved by the Sterling Institutional Review Board (sterlingirb.com; Atlanta GA, USA) and has therefore been performed in accordance with the ethical standards in the Declaration of Helsinki. Each participant was informed of the experimental procedures and associated risks before providing written informed consent.

### 2.2 Preliminary screening measurements

After providing written informed consent, subjects came to the laboratory for a screening visit to determine eligibility for participation. Measurements included body mass, height, resting heart rate, resting blood pressure, and 8‐h fasted blood glucose concentration (Contour NEXT ONE, Ascensia Diabetes Care US Inc; Parsippany, NJ). During this preliminary screening visit subjects also completed a graded exercise test on an electronically‐braked stationary cycle ergometer (Velotron, Racermate Inc.; Seattle, WA) to assess cardiorespiratory fitness (12‐lead electrocardiogram (ECG), Schiller AT‐10 Plus, Schiller America; Doral, FL), maximal heart rate (HR_max_), and peak oxygen consumption (VO_2peak_) (MOXUS, AEI Technologies; Pittsburgh, PA). The main criteria for exclusion were an abnormal resting or exercise ECG, smoking, asthma, pregnancy (self‐reported), cardiovascular, metabolic, or renal disease, or the taking of medications that may influence thermoregulatory or cardiovascular function.

### 2.3 Study design

Subjects completed two experimental trials in which they performed 90 min of cycling in a heated chamber (~33°C, 42% rh, 2.2 m/s airflow via front facing fan), either (1) with fluid replacement to maintain EUH or (2) without fluid to dehydrate to ~2%–3% of body mass loss. The conditions were chosen to elicit 2%–3% loss of body mass via sweating in a 90‐min timeframe in recreational to moderately‐trained subjects, based on previous experience and published literature (Osterberg et al., 2010; Shirreffs et al., 2007). Experimental trials were scheduled 3–14 days apart and were assigned in a randomized counterbalanced crossover design. All trials were completed July through October in Valhalla, New York. Heat‐acclimation status was not measured, but since the study was conducted in warmer months of the year subjects may have been partially to fully acclimated. However, completion of the exercise bouts in this study was unlikely to have significant effects on heat acclimation (Barnett & Maughan, 1993).

### 2.4 Experimental procedures

Subjects reported to the laboratory between 7:00 and 10:00 am for each testing day after having fasted overnight. They were instructed to eat a consistent diet, consume 2.5 L (women) or 3.2 L (men) of water, and avoid alcohol and vigorous exercise in the 24 h preceding each trial. Subjects were also instructed to consume 16 oz of water 2 h before their scheduled session to facilitate baseline EUH (i.e., subjects starting each trial with a urine specific gravity (USG) of <1.020 based on a spot sample measure).

Upon arrival to the laboratory, subjects gave investigators their diet and physical activity logs to confirm pretrial instructions were followed. Then subjects voided their bladders and collected the urine for a baseline spot sample measurement of USG (Atago Pen Refractometer, 3741‐E04 Saitama, Japan). If baseline USG was ≥1.025 the trial was rescheduled. If baseline USG was 1.020–1.024, subjects were given 16 oz of water followed by a urine sample ~ 15 min later to confirm USG <1.020. After urine assessment, the subjects' baseline nude body mass was measured in duplicate (KCC300 platform and ICS439 reader; Mettler Toledo, Columbus, OH). Then subjects sat upright for 10 min in a temperate room (21.7 ± 0.3°C, 37 ± 5%) before a fingertip capillary blood sample was obtained via a lancet. Blood Na^+^, Cl^−^, and K^+^ were immediately analyzed using an iSTAT 1 wireless point of care analyzer (Chem8+ Cartridge) (Abbott; Princeton, NJ).

After baseline assessments, the subjects went into the heat chamber and mounted an electronically‐braked stationary cycle ergometer (Velotron, Racermate Inc.; Seattle, WA). The ergometer was set at a resistance that elicited ~80% HR_max_. Heart rate was monitored using telemetry (Polar H10; Lake Success, NY). Ratings of perceived exertion (RPE) (Borg, 1982), power, and cadence were recorded every 10 min during exercise.

The 90 min of exercise consisted of three 30 min‐bouts separated by a 5‐min break to measure the subjects' nude body mass and allow them to void their bladder if needed. During the EUH trials participants drank a commercially‐available carbohydrate‐free electrolyte beverage containing 36 mmol/L sodium, 50 mmol/L chloride, and 15 mmol/L potassium (Gatorlyte Zero, manufactured by Gatorade). Fluid was consumed in a volume equivalent to 7 mL/kg body mass in the first cycling bout and 110% of body mass loss during the second and third bout. This drinking protocol was based on pilot work and designed to replace sweat and urine losses so that subjects maintained EUH throughout the trial. Subjects were not allowed to consume any fluid during the DEH trials. Based on previous studies acute intake of electrolytes during exercise would not be expected to alter sweat electrolyte concentrations (Baker et al., 2008; Hamouti et al., 2012; Nuccio et al., 2020).

**FIGURE 1:**
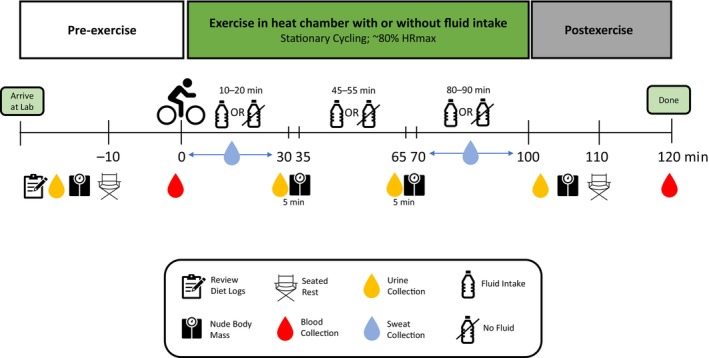
Schematic of protocol timeline.

After completion of the third exercise bout, a final nude body mass was measured in duplicate. Subjects were instructed to towel dry prior to all body mass measurements. Then subjects sat upright for 10 min in a temperate room (22.0 ± 0.2°C, 36 ± 5%) followed by collection of a postexercise fingertip capillary blood sample. Blood Na^+^, Cl^−^, and K^+^ were immediately analyzed using the iSTAT 1 wireless point of care analyzer. A schematic of the protocol procedures and timeline is shown in Figure 1.

### 2.5 Sweat collection and analyses

The standard absorbent patch method (3 M Tegaderm™ + Pad) (Baker et al., 2018) was used to collect regional sweat for this study. The forehead (FH), right scapula (SCAP), right ventral forearm (RVFA), and left ventral forearm (LVFA) were chosen to include a variety of sites across the upper body to determine the effect of hydration status on region‐specific differences in sweat electrolyte concentrations. These sites also represent a broad range in LSR and sweat Na^+^, Cl^−^, and K^+^ concentrations (Baker et al., 2018).

To assess the effect of hydration status on the change in sweat electrolyte concentrations, sweat samples were collected during the first 30‐min bout (BEGIN) and the third 30‐min bout (END) of the 90‐min cycling protocol. Immediately before exercise, the FH, SCAP, RVFA, and LVFA skin regions were cleaned with alcohol swabs and air dried. Then the first set of absorbent patches was applied to each site just before the subject mounted the bike and started exercising. An elastic net dressing (Surgilast; Derma Sciences, Princeton, NJ) was put on the RVFA and LVFA to ensure that the patch remained adhered to the skin. The first set of patches was then removed in the last ~2 min of the first 30‐min exercise bout. The skin remained uncovered during the second exercise bout. Immediately before the subjects mounted the bike for the third exercise bout, the skin was cleaned with deionized water and wiped dry, then cleaned with alcohol pads and air dried, followed by application of the second set of patches. Finally, the second set of patches was removed in the last ~2 min of the third 30‐min exercise bout. Total application time for all patches was 27.8 ± 1.4 min.

Once patches were removed, the absorbent pad was detached from the Tegaderm with clean forceps and placed in an air‐tight plastic tube (Sarstedt Salivette, Nümbrecht, Germany). Then sweat pad mass was measured to the nearest 0.001 g using an analytical balance (Mettler Toledo Balance XS204, Columbus, OH). Sweat from the absorbent patch was extracted using centrifugation, stored in sealed cryovials at −20°C, and subsequently analyzed for Na^+^, Cl^−^, and K^+^ concentrations in duplicate by ion chromatography (Dionex ICS‐6000).

### 2.6 Calculations

LSR (in mg/cm^2^/min) was determined from the mass of sweat absorbed in the pad, the pad surface area (11.9 cm^2^), and the duration of time the patch was on the skin. Whole‐body sweating rate was calculated from the change in nude body mass, corrected for fluid intake and urine loss, divided by exercise duration. Fluid intake (during the EUH trial) was calculated as the difference in drink bottle mass from pre‐ to postexercise to the nearest 0.01 g using a compact digital scale (model PG802, Mettler Toledo, Columbus, OH). Urine loss was also measured to the nearest 0.01 g using a compact digital scale (model PG5002‐S, Mettler Toledo, Columbus, OH).

### 2.7 Statistical analyses

Statistical analyses were performed using Minitab 19 Statistical Software (Minitab, Inc., State College, PA, USA). G*Power 3 was used for the power calculation. Based on a mean difference between EUH and DEH of 10 mmol/L for sweat Na^+^ concentration in the study by Morgan et al. (2004), an estimated correlation coefficient of 0.75, and an effect size estimate of 0.89, a sample size of *n* = 12 was needed to detect a significant difference with a two‐sided paired *t*‐test (alpha = 0.05, Power = 0.80).

Mean ± SD values and individual data for LSR (Table S1, Figure S1), sweat electrolyte concentrations (Tables S2–S5, Figures S2–S5), and blood electrolyte concentrations (Table S6) are shown in the Supplemental Material. Delta values were calculated as END minus BEGIN for all variables and used for analyses. Paired *t*‐tests were used to determine the effect of hydration status (EUH vs. DEH) on the change in LSR, sweat Na^+^, Cl^−^, K^+^, concentrations, Na^+^/K^+^ concentration ratio, and blood Na^+^, Cl^−^, K^+^ concentrations. Wilcoxon signed‐rank tests were used for nonnormally distributed data. Shapiro–Wilk tests were used to assess the normality of the residuals. The significance level was set at *p* < 0.05. Data are presented as mean ± SD for normally distributed data or median ± IQR for nonnormally distributed data (RVFA and LVFA sweat K^+^, SCAP sweat Cl^−^, and RVFA LSR).

Because of low sweat rates in the first 30 min of exercise, three subjects had insufficient sample volume for sweat electrolyte analysis. Therefore, the sample size for the final analysis of sweat electrolyte concentrations at all sites was *n* = 12, while the final sample size for the LSR analyses was *n* = 15. Six of the 15 subjects had missing blood data from at least one time point due to difficulty in obtaining sufficient sample via finger prick for iSTAT analysis (after a maximum of 2 attempts). Therefore, the final sample size for analyses of blood electrolyte concentrations was *n* = 9.

## 3 RESULTS

### 3.1 Descriptive data

The participants VO_2peak_ during the preliminary screening was 47.6 ± 7.0 mL/kg/min. Table 1 shows the descriptive data for EUH and DEH trials. Baseline nude body mass (*p* = 0.26) and USG (*p* = 0.45) did not differ between the EUH and DEH trials. During exercise, whole body sweat loss (*p* = 0.10), WBSR (*p* = 0.10), and urine loss (*p* = 0.84) did not differ between the EUH and DEH trials. In addition, mean power output (*p* = 0.33), air temperature (*p* = 0.13), and relative humidity (*p* = 0.15) during exercise were not different between trials. However, mean RPE (*p* = 0.01) and %HR_max_ (*p* < 0.01) during exercise were significantly higher during the DEH than the EUH trials. As expected, net fluid balance (% change in body mass) was significantly different between EUH and DEH trials (*p* < 0.0001). Further, the percentage change in body mass loss was significantly greater at END vs. BEGIN of the DEH trial (*p* < 0.0001). Subjects' net fluid balance after the third exercise bout ranged from 1.60% to 2.87% in the DEH trial. Total fluid intake during the EUH trial was 1.72 ± 0.33 L.

**TABLE 1:** Descriptive data.

	Euhydration	Dehydration
Baseline nude body mass (kg)	71.73 ± 14.76	71.54 ± 14.39
Baseline urine specific gravity	1.007 ± 0.004	1.006 ± 0.005
Whole body sweating rate (L/h)	0.86 ± 0.22	0.92 ± 0.24
Whole body sweat loss (L)	1.43 ± 0.36	1.53 ± 0.39
Urine loss (g)	193 ± 79	184 ± 183
Net fluid balance after 1st exercise bout (% change in body mass)*	−0.10 ± 0.22	−0.92 ± 0.30
Net fluid balance after 3rd exercise bout (% change in body mass)*	0.14 ± 0.13	−2.40 ± 0.38^#^
Rating of perceived exertion (6–20 scale)*	12.6 ± 2.0	13.5 ± 2.2
Power output (Watts)	138 ± 32	139 ± 30
Percentage of maximal heart rate (%)*	80 ± 5	83 ± 5
Air temperature (°C)	32.5 ± 0.6	32.7 ± 0.6
Relative humidity (%)	43 ± 3	41 ± 2

*Note*: Values are mean ± SD; *N* = 15; *p < 0.05, Euhydration vs. Dehydration; #*p* < 0.05, Net fluid balance within dehydration trial.

### 3.2 Local sweating rate

**FIGURE 2:**
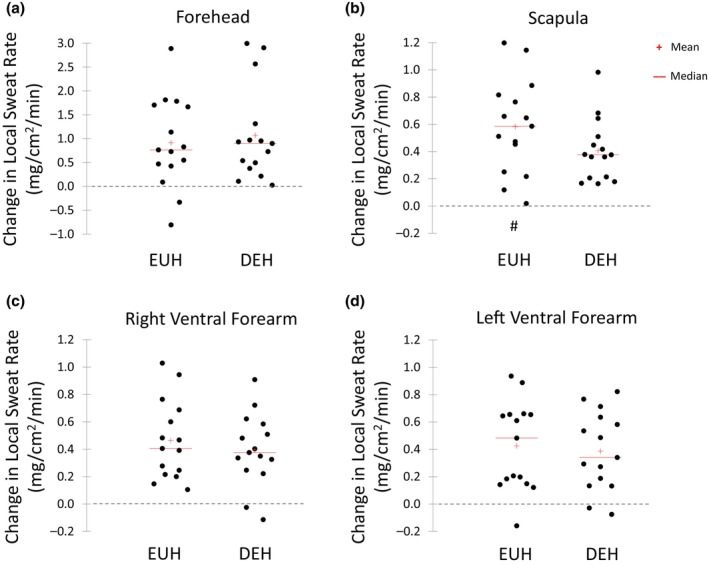
Change in local sweating rate (mg/cm^2^/min) from the beginning to end of exercise at the Forehead (a), Scapula (b), Right Ventral Forearm (c), and Left Ventral Forearm (d) during the euhydration (EUH) and dehydration (DEH) trials. ^#^The increase in Scapula LSR was significantly greater in the EUH trial than the DEH trial (paired *t*‐test, *p* = 0.02).

Figure 2 shows scattergrams for the changes in LSR from BEGIN to END of exercise for all sites. There were no significant differences between EUH and DEH in the change in LSR at the FH (0.91 ± 0.95 vs. 1.07 ± 0.98 mg/cm^2^/min, *p* = 0.37), RVFA (0.41 ± 0.47 vs. 0.38 ± 0.34 mg/cm^2^/min, *p* = 0.59), or LVFA (0.43 ± 0.32 vs. 0.39 ± 0.29 mg/cm^2^/min, *p* = 0.49). The increase in SCAP LSR from BEGIN to END was significantly greater in the EUH trial than the DEH trial (0.58 ± 0.35 vs 0.41 ± 0.23 mg/cm^2^/min, *p* = 0.02).

### 3.3 Sweat electrolyte concentrations

**FIGURE 3:**
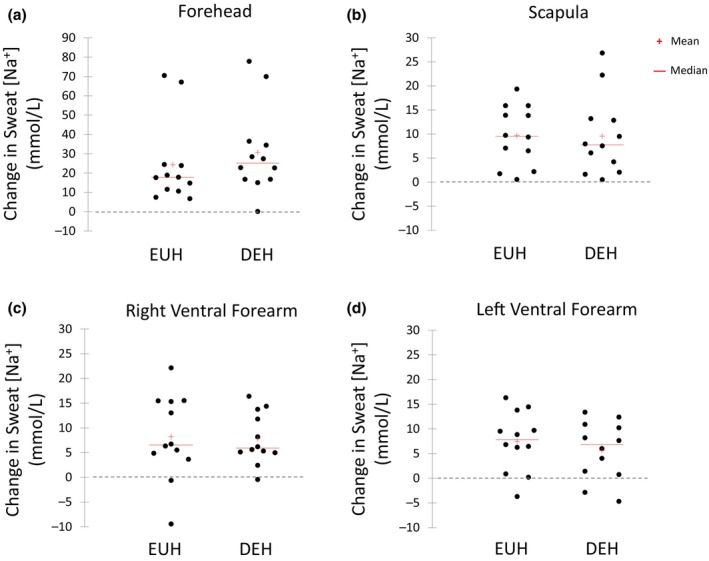
Change in sweat sodium concentration (mmol/L) from the beginning to end of exercise at the Forehead (a), Scapula (b), Right Ventral Forearm (c), and Left Ventral Forearm (d) during the euhydration (EUH) and dehydration (DEH) trials.

**FIGURE 4:**
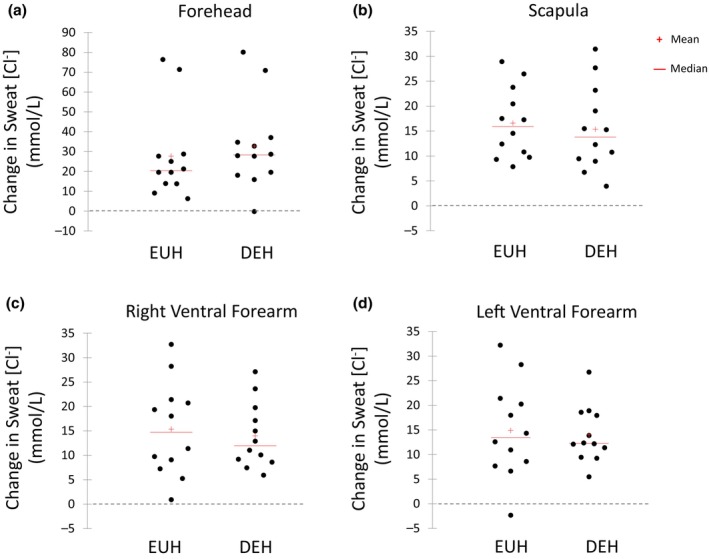
Change in sweat chloride concentration (mmol/L) from the beginning to end of exercise at the Forehead (a), Scapula (b), Right Ventral Forearm (c), and Left Ventral Forearm (d) during the euhydration (EUH) and dehydration (DEH) trials.

**FIGURE 5:**
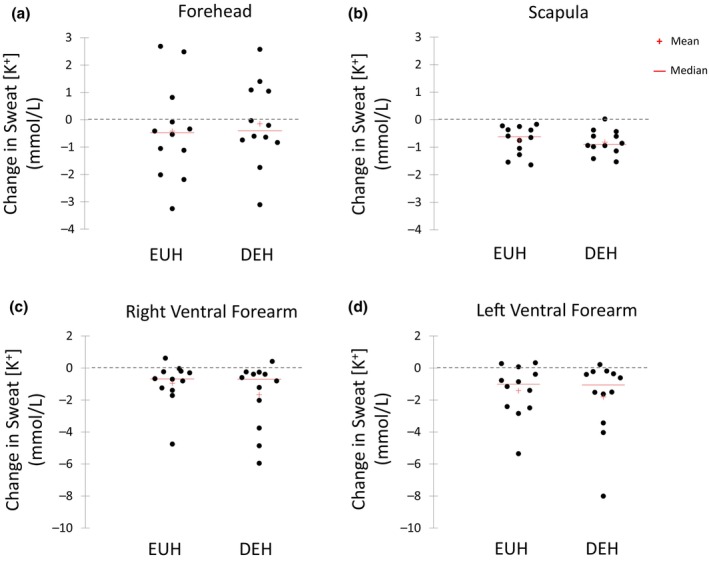
Change in sweat potassium concentration (mmol/L) from the beginning to end of exercise at the Forehead (a), Scapula (b), Right Ventral Forearm (c), and Left Ventral Forearm (d) during the euhydration (EUH) and dehydration (DEH) trials.

**FIGURE 6:**
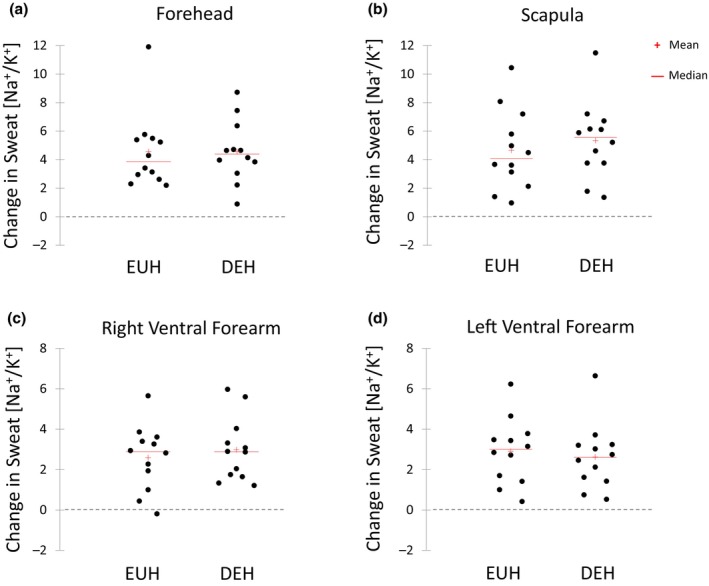
Change in sweat sodium/potassium concentration ratio from the beginning to end of exercise at the Forehead (a), Scapula (b), Right Ventral Forearm (c), and Left Ventral Forearm (d) during the euhydration (EUH) and dehydration (DEH) trials.

Figures 3, 4, 5, 6 show the scattergrams for absolute changes in sweat concentrations of Na^+^ (Figure 3), Cl^−^ (Figure 4), K^+^ (Figure 5), and the Na^+^/K^+^ concentration ratio (Figure 6) from BEGIN to END for all sites. There were no significant differences between EUH and DEH in sweat Na^+^ concentration changes at the FH (24.3 ± 21.5 vs. 30.8 ± 22.4 mmol/L, *p* = 0.07), SCAP (9.7 ± 6.2 vs. 9.6 ± 8.2 mmol/L, *p* = 0.95), RVFA (8.2 ± 8.6 vs. 7.8 ± 5.2 mmol/L, *p* = 0.84), or LVFA (7.5 ± 6.0 vs. 5.6 ± 5.9 mmol/L, *p* = 0.29). Changes in sweat Cl^−^ concentration were also not significantly different between EUH and DEH trials at the FH (27.7 ± 22.7 vs. 32.8 ± 22.5 mmol/L, *p* = 0.14), SCAP (15.9 ± 12.9 vs. 13.8 ± 13.1 mmol/L, *p* = 0.31), RVFA (15.3 ± 9.6 vs. 14.0 ± 6.7 mmol/L, *p* = 0.36), or LVFA (14.9 ± 9.7 vs. 14.0 ± 5.7 mmol/L, *p* = 0.62). Furthermore, there were no significant differences between EUH and DEH in sweat K^+^ concentration changes at the FH (−0.4 ± 1.8 vs. −0.2 ± 1.5 mmol/L, *p* = 0.48), SCAP (−0.7 ± 0.5 vs. −0.8 ± 0.4 mmol/L, *p* = 0.74), RVFA (−0.7 ± 1.2 vs. −0.7 ± 3.0 mmol/L, *p* = 0.26), or LVFA (−1.0 ± 2.43 vs. −1.1 ± 2.7 mmol/L, *p* = 0.99). Finally, there were no significant differences between EUH and DEH in sweat Na^+^/K^+^ concentration ratio changes at the FH (4.6 ± 2.7 vs. 4.6 ± 2.2, *p* = 0.99), SCAP (4.7 ± 2.8 vs. 5.3 ± 2.7, *p* = 0.30), RVFA (2.6 ± 1.6 vs. 3.0 ± 1.6, *p* = 0.34), or LVFA (2.9 ± 1.6 vs. 2.6 ± 1.6, *p* = 0.54).

A summary of the number of subjects who experienced an increase or decrease in LSR and sweat electrolyte concentrations from the beginning to end of exercise is shown in the supplemental material (Table S7). LSR increased from the beginning to end of exercise in most subjects during the EUH (FH: *n* = 13/15, SCAP: *n* = 15/15, RVFA: *n* = 15/15, LVFA: *n* = 14/15) and DEH (FH: *n* = 15/15, SCAP: *n* = 15/15, RVFA: *n* = 13/15, LVFA: *n* = 13/15) trials. In addition, sweat Na^+^, Cl^+^, and Na^+^/K^+^ concentrations increased from the beginning to end of exercise in most subjects during the EUH and DEH trials. Specifically, for sweat Na^+^ the number of subjects with increased concentrations was *n* = 12/12, *n* = 12/12, *n* = 10/12, and *n* = 11/12 for FH, SCAP, RVFA, and LFVA, respectively during the EUH trials and *n* = 12/12, *n* = 12/12, *n* = 11/12, and *n* = 10/12 for FH, SCAP, RVFA, and LFVA, respectively during the DEH trials. For sweat Cl^−^ the number of subjects with increased concentrations was *n* = 12/12, *n* = 12/12, *n* = 12/12, and *n* = 11/12 for FH, SCAP, RVFA, and LFVA, respectively during the EUH trials and *n* = 11/12, *n* = 12/12, *n* = 12/12, and *n* = 12/12 for FH, SCAP, RVFA, and LFVA, respectively during the DEH trials. For sweat Na^+^/K^+^ the number of subjects with increased concentrations was *n* = 12/12, *n* = 12/12, *n* = 11/12, and *n* = 12/12 for FH, SCAP, RVFA, and LFVA, respectively during the EUH trials and *n* = 12/12 for all sites during the DEH trials. On the other hand, sweat K^+^ concentration decreased from the beginning to end of exercise in most subjects during the EUH (FH: *n* = 9/12, SCAP: *n* = 12/12, RVFA: *n* = 11/12, LVFA: *n* = 9/12) and DEH (FH: *n* = 8/12, SCAP: *n* = 11/12, RVFA: *n* = 11/12, LVFA: *n* = 11/12) trials.

### 3.4 Blood electrolyte concentrations

The increase in blood Na^+^ concentration (3.3 ± 1.9 vs. 1.0 ± 1.5 mmol/L, *p* < 0.01) and Cl^−^ concentration (4.0 ± 4.0 vs. 2.0 ± 5.0 mmol/L, *p* = 0.02) from pre‐ to postexercise was significantly greater in the DEH trial than the EUH trial. There was no difference between EUH and DEH for the change in blood K^+^ concentration (0.9 ± 0.8 vs. 0.6 ± 0.7 mmol/L, *p* = 0.26).

## 4 DISCUSSION

The purpose of this study was to compare the effect of ~2.4% DEH versus EUH on the acute changes in sweat Na^+^, Cl^−^, and K^+^ concentrations during exercise. This was an important question to address since one of the proposed applications for on‐body sweat analytics is real‐time monitoring of hydration status during exercise. The main finding was that changes in sweat electrolyte concentrations during 90 min of exercise in the heat were not significantly influenced by hydration status in recreational to moderately‐trained male and female athletes. These results suggest that sweat electrolyte concentrations are not a useful biomarker for detecting DEH during exercise‐heat stress.

Previous studies have suggested that changes in sweat electrolyte concentrations can serve as an indicator of DEH (Gao et al., 2016; Wang et al., 2022; Yang, Sun, et al., 2023) since sweat Na^+^ concentration has been shown to increase (Gao et al., 2016; Yang, Sun, et al., 2023), and K^+^ concentration decreased (Yang, Sun, et al., 2023) over time when subjects were dehydrated during exercise. A novel aspect of the present study was the crossover design, which allowed for the comparison of sweat electrolyte changes between a DEH condition and a control EUH trial. While most subjects in the present study did experience an increase in Na^+^ and Cl^−^ concentrations and a decrease in K^+^ concentration from the beginning to end of exercise, these changes did not differ between EUH and DEH trials. This finding suggests that time course changes in sweat electrolyte concentrations during exercise are due to factors unrelated to hydration status per se. As discussed previously, other factors that may impact acute changes in sweat electrolyte concentrations include LSR and skin surface mineral content (Buono et al., 2008; Ely et al., 2011; Verissimo et al., 2007).

As expected, most (at least 13 of 15) subjects experienced an increase in LSR from the beginning to the end of exercise at all body sites. A lower LSR occurred at the beginning of exercise, likely because of the time required to initiate and ramp up the sweating response in the first 30 min of exercise‐heat stress. In turn, the increase in LSR was associated with an increase in sweat Na^+^ and Cl^−^ concentrations (for at least 10 of 12 subjects) from the beginning to the end of exercise at all sites. This result is consistent with several previous studies showing the direct association between LSR and sweat Na^+^ and Cl^−^ concentrations (Baker et al., 2019; Buono et al., 2007, 2008; Johnson et al., 1944). On the other hand, sweat K^+^ concentration decreased from the beginning to the end of exercise in most (at least 8 of 12) participants, especially on the SCAP, RVFA, and LVFA. The appearance of higher concentrations of K^+^ in the first 30 min of sweat sampling may be due to the presence of K^+^ in the stratum corneum, while subsequently the lower sweat K^+^ concentration at the end of exercise may be a result of flushing away the contaminants by further sweating (Ely et al., 2011; Verissimo et al., 2007). It is important to note that this skin surface potassium may show up in initial sweat samples despite cleaning the skin with alcohol before placing the patch, as previous studies have shown thorough abrasion of the skin is needed to remove desquamated skin cells and associated minerals (Ely et al., 2011).

In the present study, blood Na^+^ and Cl^−^ concentrations were higher in the DEH versus EUH trials at the end of exercise, which is an expected effect of DEH‐induced hemoconcentration. However, this was not associated with a difference in sweat electrolyte concentrations between DEH and EUH trials. Our results are in contrast to the findings of Morgan et al. (2004), who reported a significant effect of hydration status on both serum and sweat Na^+^ and Cl^−^ concentrations during 120 min of cycling. However, the differences in serum Na^+^ and Cl^−^ concentrations between DEH and EUH trials reported by Morgan et al. (2004) were too small (3 mmol/L) to account for the differences in sweat Na^+^ and Cl^−^ concentrations (10 mmol/L and 5 mmol/L, respectively). Other mechanisms to explain the previously reported higher sweat electrolyte concentrations with DEH, such as greater plasma aldosterone or sympathetic nervous activity, have been suggested (Morgan et al., 2004). However, a plausible explanation could be measurement or physiological variability, since the between trial differences reported by Morgan et al. (2004) were within the expected day‐to‐day variation in forearm sweat Na^+^ and Cl^−^ concentrations (~12%) (Baker et al., 2018). Taken together, the data suggest that sweat electrolyte concentrations are not a reliable indicator of hydration status during exercise.

Another novel aspect of this study was the analyses of sweat electrolyte concentration changes from multiple body regions, as most aforementioned studies collected sweat from only one site (Morgan et al., 2004; Walsh et al., 1994; Yang, Sun, et al., 2023). Multiple sites were chosen for this study to represent a broad range in LSR and sweat Na^+^, Cl^−^, and K^+^ concentrations (Baker et al., 2018). Moreover, including the FH (Gao et al., 2016) and forearms (Morgan et al., 2004; Walsh et al., 1994) allowed for comparisons to previous studies assessing sweat electrolytes and hydration status. Nonetheless, the results of the present study were the same across all body sites, suggesting no regional or bilateral differences in the effect of hydration status on sweat electrolyte concentrations.

In conclusion, this randomized counterbalanced crossover study found that ~2.4% DEH did not significantly influence acute changes in sweat electrolyte concentrations during exercise‐heat stress. Although sweat Na^+^ and Cl^−^ concentrations increased and sweat K^+^ concentration decreased from the beginning to the end of 90 min of exercise, the changes did not differ between EUH and DEH conditions. The findings were consistent across all body sites tested (FH, SCAP, RVFA, and LVFA). These results suggest that sweat electrolyte concentrations are not a useful biomarker for detecting mild DEH during exercise‐heat stress.

## FUNDING INFORMATION

This study was funded by the Gatorade Sports Science Institute, a division of PepsiCo R&D.

## CONFLICT OF INTEREST STATEMENT

L.B.B., M.O., J.R.M., S.A., P.J.D.D. and J.M.H are employed by PepsiCo R&D. The views expressed in this manuscript are those of the authors and do not necessarily reflect the position or policy of PepsiCo, Inc.

## ETHICS STATEMENT

This study was approved by the Sterling Institutional Review Board (sterlingirb.com; Atlanta GA, USA) and has therefore been performed in accordance with the ethical standards in the Declaration of Helsinki.

## PATIENT CONSENT STATEMENT

Each participant was informed of the experimental procedures and associated risks before providing written informed consent.

## CLINICAL TRIAL REGISTRATION

clinicaltrials.gov NCT06044610.

## Supporting information


Figure S1.

Table S2.

## Data Availability

The data that supports the findings of this study are available from the authors upon reasonable request.
