# Supplementary material for: Mild dehydration does not alter acute changes in sweat electrolyte concentrations during exercise
Source: Physiol Rep. 2024 Sep 18;12(18):e16174. doi: 10.14814/phy2.16174 (PMC11410553; doi:10.14814/phy2.16174)

Supplemental Figures

Mild Dehydration Does Not Alter Acute Changes in Sweat Electrolyte Concentrations during Exercise

Lindsay B. Baker[1*], Michal Ozga[1], James R. Merritt[1], Shelby Alfred[1], Peter John D. De Chavez[2], J. Matthew Hinkley[1]

[1]Gatorade Sports Science Institute, PepsiCo R&D Life Sciences, Valhalla, NY, USA

[2]Data Science & Analytics, PepsiCo R&D, Plano, TX, USA

*Corresponding Author
Lindsay B. Baker
Gatorade Sports Science Institute
PepsiCo R&D Life Sciences
50 E. Stevens Ave.
Valhalla, NY, 10595
Email: lindsay.baker@pepsico.com

Supplemental Figure 1. Local Sweat Rate

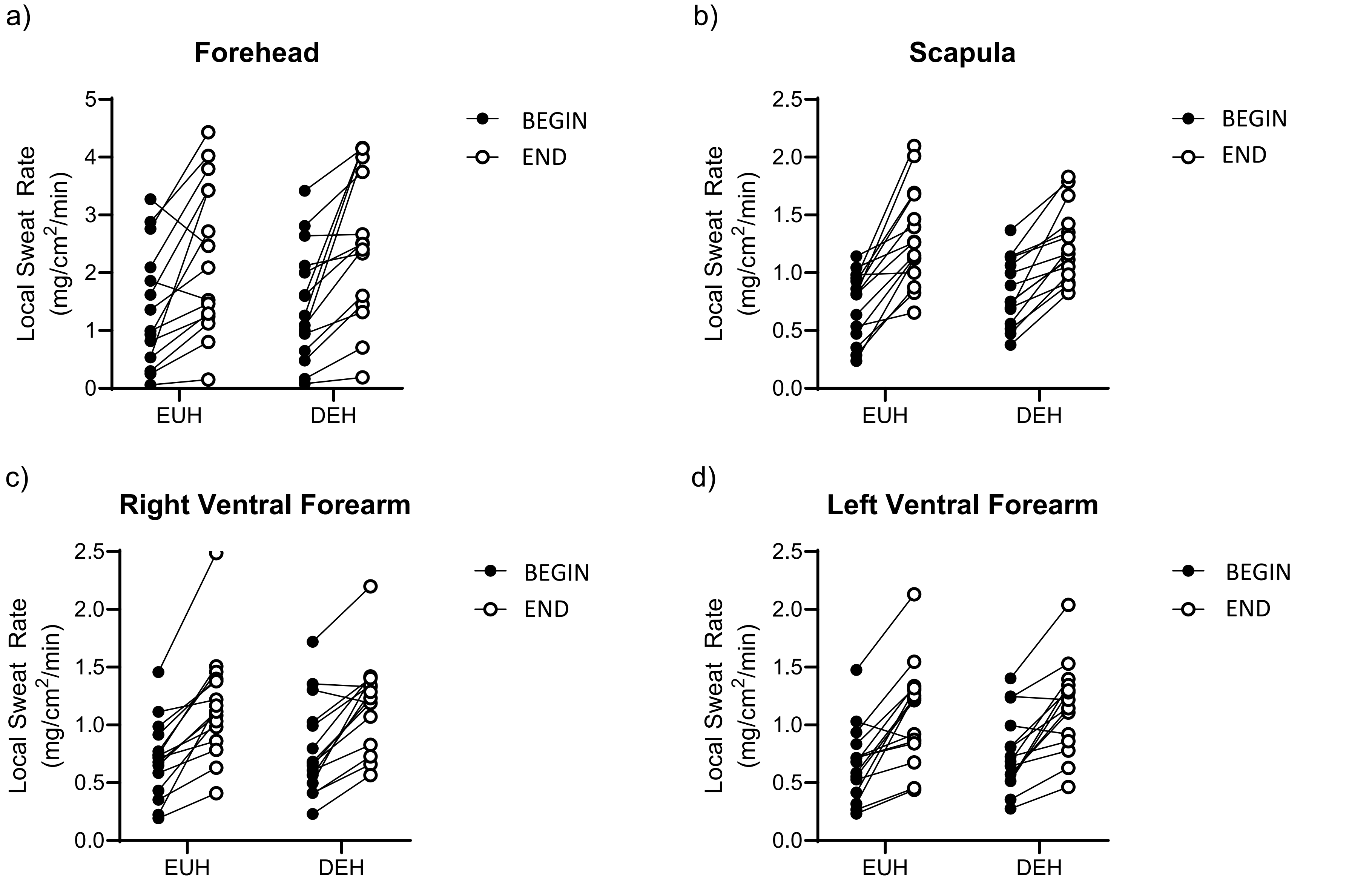

Supplemental Figure 2. Sweat Sodium ([Na+])

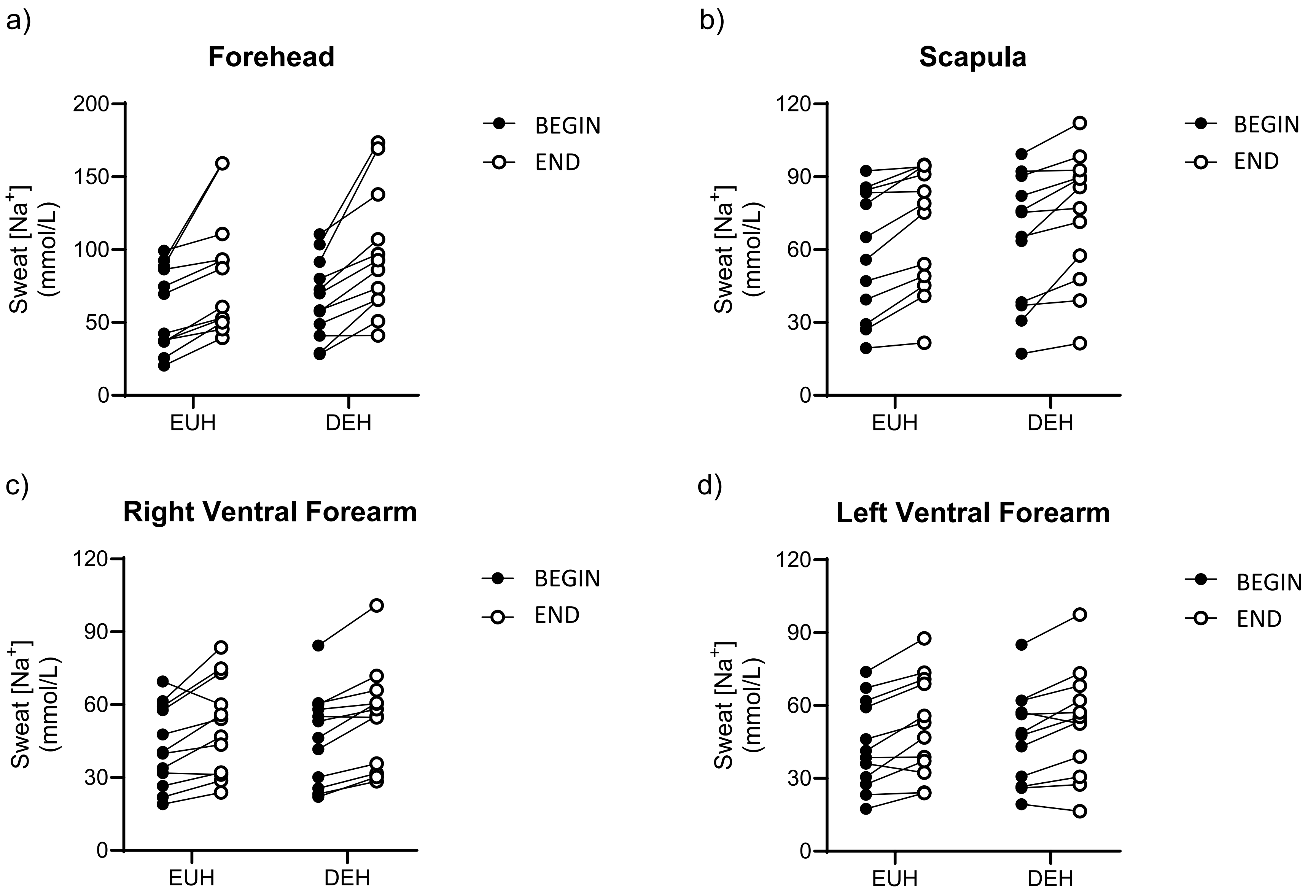

Supplemental Figure 3. Sweat Chloride Concentration ([Cl$^-$])

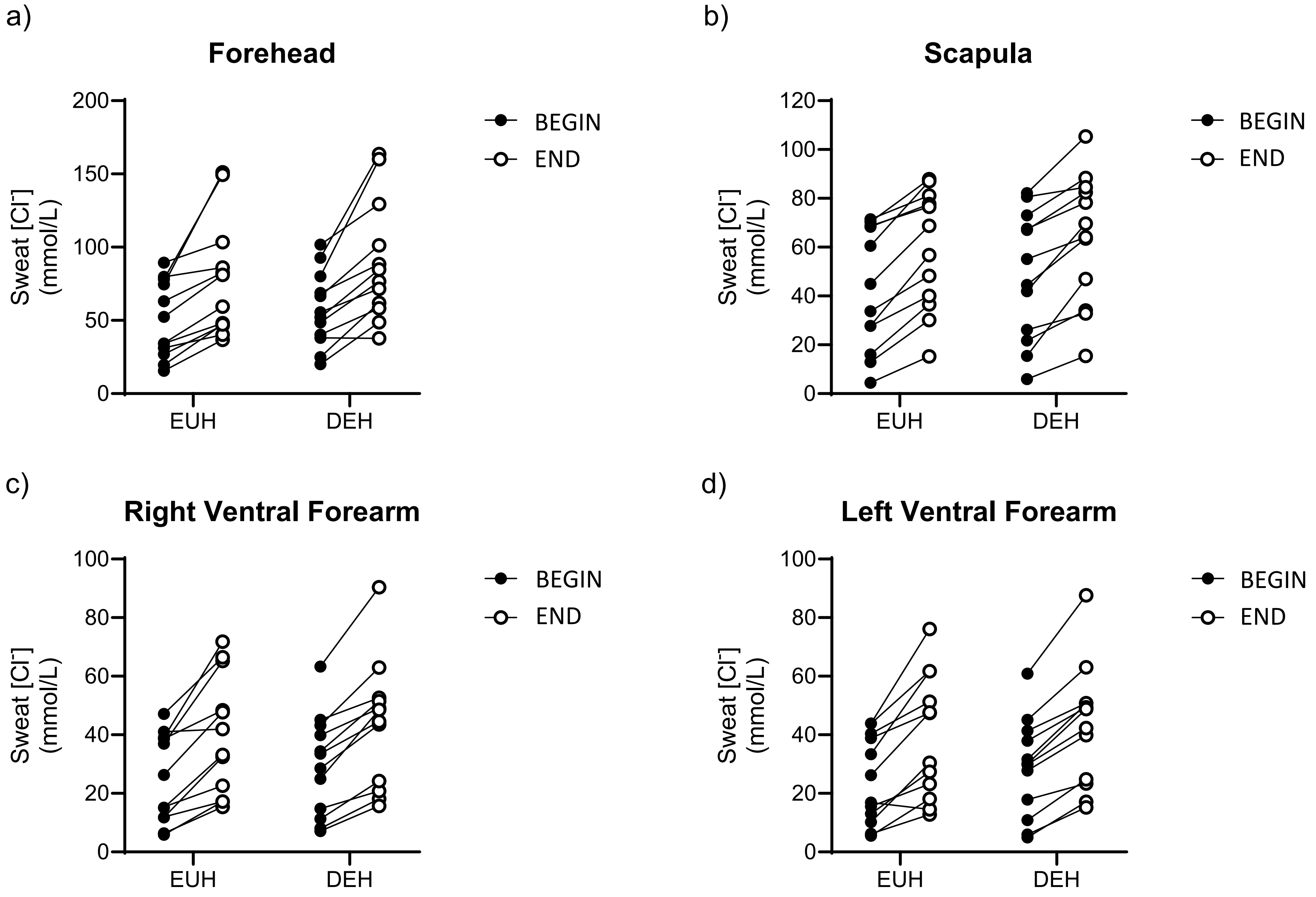

Supplemental Figure 4. Sweat Potassium Concentration ([K+])

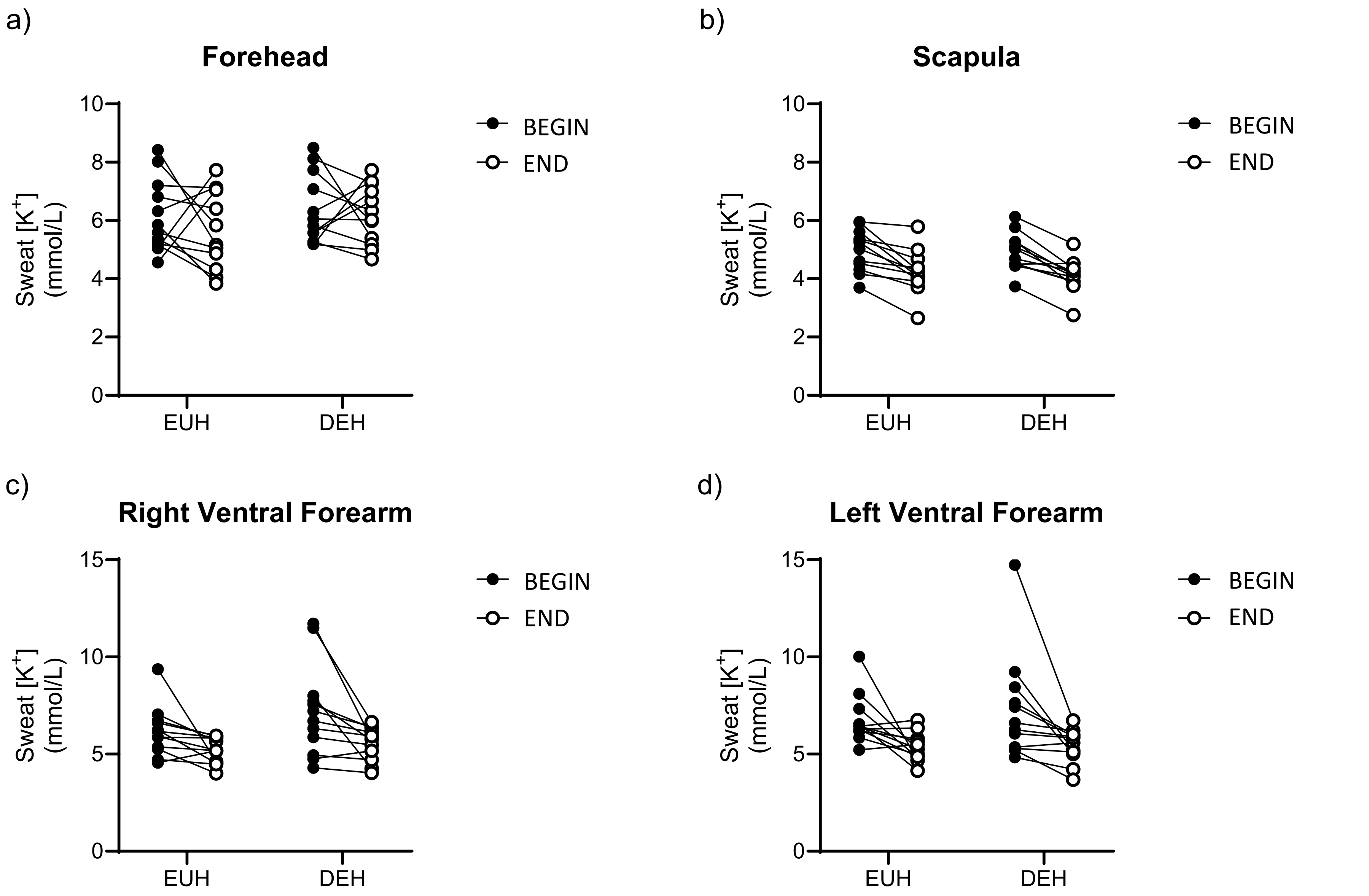

Supplemental Figure 5. Sweat Sodium:Potassium Concentration Ratio ([Na^+:K^+])

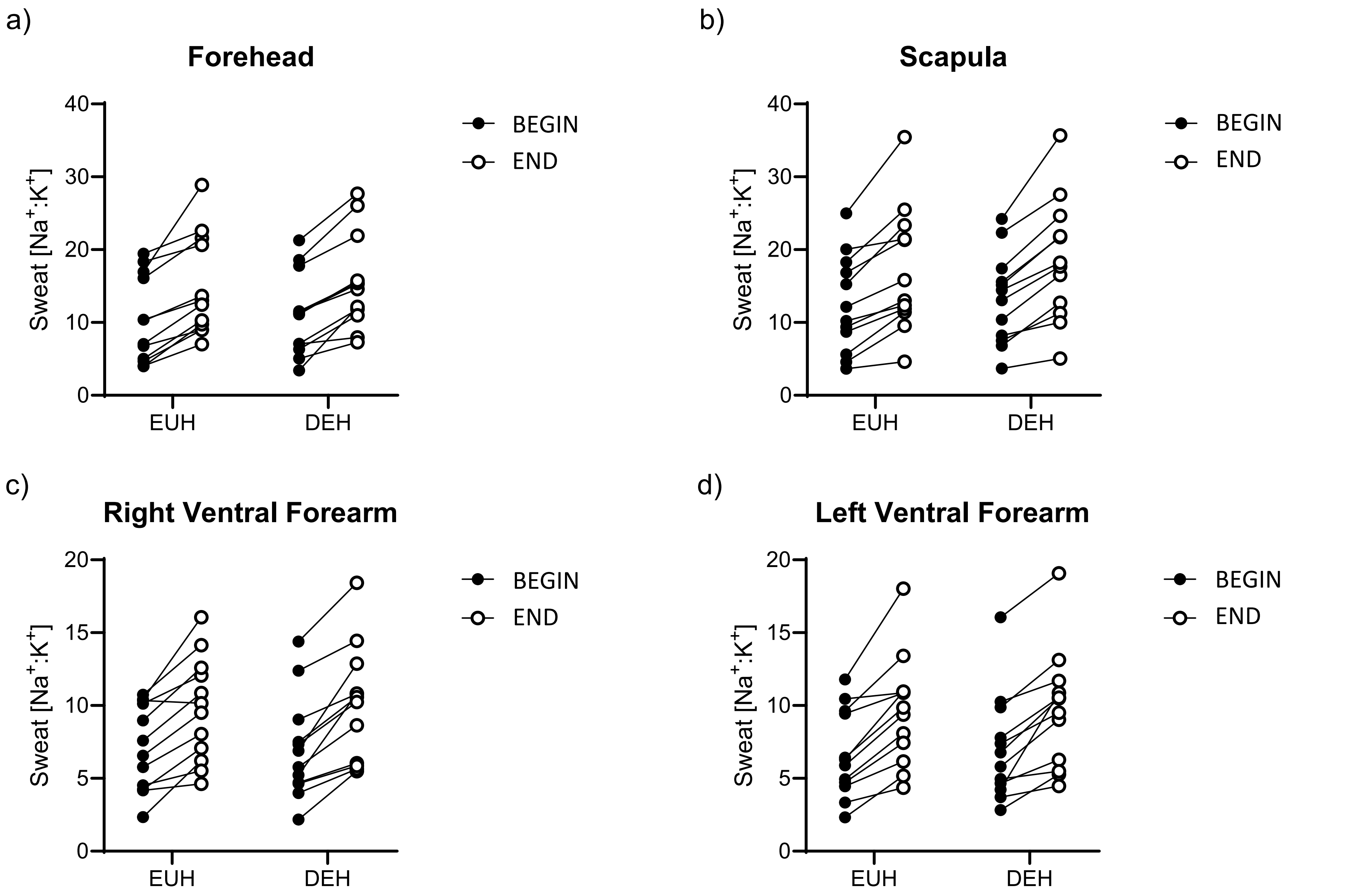

Supplement: Supplementary file 1 — Figure S1. [file PHY2-12-e16174-s002.pdf]
