# Supplementary material for: Mild dehydration does not alter acute changes in sweat electrolyte concentrations during exercise
Source: Physiol Rep. 2024 Sep 18;12(18):e16174. doi: 10.14814/phy2.16174 (PMC11410553; doi:10.14814/phy2.16174)
Supplement: Supplementary file 2 — Table S2. [file PHY2-12-e16174-s001.docx]

**Supplemental Tables**

**Mild Dehydration Does Not Alter Acute Changes in Sweat Electrolyte Concentrations during Exercise**

Lindsay B. Baker^1*^, Michal Ozga^1^, James R. Merritt^1^, Shelby Alfred^1^, Peter John D. De Chavez^2^, J. Matthew Hinkley^1^

^1^Gatorade Sports Science Institute, PepsiCo R&D Life Sciences, Valhalla, NY, USA

^2^Data Science & Analytics, PepsiCo R&D, Plano, TX, USA

*Corresponding Author

Lindsay B. Baker

Gatorade Sports Science Institute

PepsiCo R&D Life Sciences

50 E. Stevens Ave.

Valhalla, NY, 10595

Email: [lindsay.baker@pepsico.com](mailto:lindsay.baker@pepsico.com)

**Supplemental Table 1. Local sweating rates**

|  | Forehead | | Scapula | | Right Ventral Forearm | | Left Ventral Forearm | |
| --- | --- | --- | --- | --- | --- | --- | --- | --- |
|  | EUH | DEH | EUH | DEH | EUH | DEH | EUH | DEH |
| BEGIN | 1.35 ± 1.03 | 1.46 ± 0.99 | 0.73±0.29 | 0.84±0.30 | 0.70±0.34 | 0.79±0.41 | 0.67±0.32 | 0.76±0.33 |
| END | 2.27 ± 1.31 | 2.53 ± 1.31 | 1.31±0.42 | 1.24±0.32 | 1.17±0.48 | 1.19±0.40 | 1.09±0.44 | 1.14±0.39 |

Values are mean±SD in mg/cm^2^/min. EUH, euhydration; DEH, dehydration; BEGIN, 0-30 min of exercise; END, 60-90 min of exercise

**Supplemental Table 2. Sweat sodium concentrations**

|  | Forehead | | Scapula | | Right Ventral Forearm | | Left Ventral Forearm | |
| --- | --- | --- | --- | --- | --- | --- | --- | --- |
|  | EUH | DEH | EUH | DEH | EUH | DEH | EUH | DEH |
| BEGIN | 59.4±28.6 | 66.0±27.2 | 59.0±26.1 | 64.0±27.0 | 42.4±16.7 | 46.7±18.9 | 43.6±18.3 | 47.1±19.1 |
| END | 83.7±42.0 | 96.7±43.5 | 68.7±25.3 | 73.5±26.9 | 50.7±19.8 | 54.5±20.9 | 51.1±20.8 | 52.7±22.2 |

Values are mean±SD in mmol/L. EUH, euhydration; DEH, dehydration; BEGIN, 0-30 min of exercise; END, 60-90 min of exercise

**Supplemental Table 3. Sweat chloride concentrations**

|  | Forehead | | Scapula | | Right Ventral Forearm | | Left Ventral Forearm | |
| --- | --- | --- | --- | --- | --- | --- | --- | --- |
|  | EUH | DEH | EUH | DEH | EUH | DEH | EUH | DEH |
| BEGIN | 50.0±26.1 | 57.4±25.5 | 42.3± 24.9 | 48.4±26.4 | 24.6±15.2 | 29.5±17.2 | 24.5±14.9 | 28.7±16.7 |
| END | 77.7±39.9 | 90.2±41.3 | 58.8± 24.5 | 63.8±26.7 | 40.0±20.3 | 43.5±21.5 | 39.4±21.1 | 42.7±20.8 |

Values are mean±SD in mmol/L. EUH, euhydration; DEH, dehydration; BEGIN, 0-30 min of exercise; END, 60-90 min of exercise

**Supplemental Table 4. Sweat potassium concentrations**

|  | Forehead | | Scapula | | Right Ventral Forearm | | Left Ventral Forearm | |
| --- | --- | --- | --- | --- | --- | --- | --- | --- |
|  | EUH | DEH | EUH | DEH | EUH | DEH | EUH | DEH |
| BEGIN | 6.1±1.2 | 6.4±1.2 | 5.0±0.7 | 4.9±0.7 | 6.2±1.3 | 7.2±2.4 | 6.8±1.2 | 7.3±2.7 |
| END | 5.7±1.4 | 6.2±1.0 | 4.2±0.7 | 4.1±0.6 | 5.2±0.7 | 5.5±0.9 | 5.3±0.7 | 5.4±0.9 |

Values are mean±SD in mmol/L. EUH, euhydration; DEH, dehydration; BEGIN, 0-30 min of exercise; END, 60-90 min of exercise

**Supplemental Table 5. Sweat sodium/potassium concentration ratios**

|  | Forehead | | Scapula | | Right Ventral Forearm | | Left Ventral Forearm | |
| --- | --- | --- | --- | --- | --- | --- | --- | --- |
|  | EUH | DEH | EUH | DEH | EUH | DEH | EUH | DEH |
| BEGIN | 10.3±5.9 | 11.0±5.7 | 12.5±6.7 | 13.2±6.3 | 7.1±2.9 | 7.0±3.5 | 6.6±3.0 | 7.0±3.7 |
| END | 14.8±6.9 | 15.6±6.5 | 17.1±8.5 | 18.6±8.4 | 9.7±3.6 | 10.0±4.0 | 9.5±3.8 | 9.7±4.1 |

Values are mean±SD. EUH, euhydration; DEH, dehydration; BEGIN, 0-30 min of exercise; END, 60-90 min of exercise

**Supplemental Table 6. Blood electrolyte concentrations**

|  | Sodium (mmol/L) | | Chloride (mmol/L) | | Potassium (mmol/L) | |
| --- | --- | --- | --- | --- | --- | --- |
|  | EUH | DEH | EUH | DEH | EUH | DEH |
| PRE-EXERCISE | 141±2 | 142±2 | 107±1.4 | 107±1.4 | 4.9±0.4 | 4.9±0.4 |
| POST-EXERCISE | 142± 2 | 145±2 | 106±9.0 | 112±2.3 | 5.8±0.6 | 5.5±0.6 |

Values are mean±SD. EUH, euhydration; DEH, dehydration

**Supplemental Table 7. Number of subjects who experienced an increase or decrease in local sweat rate and sweat electrolyte concentrations from the beginning to end of exercise.**

|  | **EUHYDRATION** | | **DEHYDRATION** | |
| --- | --- | --- | --- | --- |
|  | **Increase** | **Decrease** | **Increase** | **Decrease** |
| **FOREHEAD** |  |  |  |  |
| Local sweat rate (n=15) | 13 | 2 | 15 | 0 |
| Sweat sodium concentration (n=12) | 12 | 0 | 12 | 0 |
| Sweat chloride concentration (n=12) | 12 | 0 | 11 | 1 |
| Sweat potassium concentration (n=12) | 3 | 9 | 4 | 8 |
| Sweat sodium/potassium ratio (n=12) | 12 | 0 | 12 | 0 |
|  |  |  |  |  |
| **SCAPULA** |  |  |  |  |
| Local sweat rate (n=15) | 15 | 0 | 15 | 0 |
| Sweat sodium concentration (n=12) | 12 | 0 | 12 | 0 |
| Sweat chloride concentration (n=12) | 12 | 0 | 12 | 0 |
| Sweat potassium concentration (n=12) | 0 | 12 | 1 | 11 |
| Sweat sodium/potassium ratio (n=12) | 12 | 0 | 12 | 0 |
|  |  |  |  |  |
| **RIGHT VENTRAL FOREARM** |  |  |  |  |
| Local sweat rate (n=15) | 15 | 0 | 13 | 2 |
| Sweat sodium concentration (n=12) | 10 | 2 | 11 | 1 |
| Sweat chloride concentration (n=12) | 12 | 0 | 12 | 0 |
| Sweat potassium concentration (n=12) | 1 | 11 | 1 | 11 |
| Sweat sodium/potassium ratio (n=12) | 11 | 1 | 12 | 0 |
|  |  |  |  |  |
| **LEFT VENTRAL FOREARM** |  |  |  |  |
| Local sweat rate (n=15) | 14 | 1 | 13 | 2 |
| Sweat sodium concentration (n=12) | 11 | 1 | 10 | 2 |
| Sweat chloride concentration (n=12) | 11 | 1 | 12 | 0 |
| Sweat potassium concentration (n=12) | 3 | 9 | 1 | 11 |
| Sweat sodium/potassium ratio (n=12) | 12 | 0 | 12 | 0 |
